# Coexistence of benign struma ovarii, pseudo-Meigs’ syndrome and elevated serum CA 125: Case report and review of the literature

**DOI:** 10.3892/ol.2015.2927

**Published:** 2015-02-03

**Authors:** CHENGJUAN JIN, RUIYING DONG, HUALEI BU, MINGYUAN YUAN, YOUZHONG ZHANG, BEIHUA KONG

**Affiliations:** 1 Department of Obstetrics and Gynecology, Qilu Hospital, Shandong University, Jinan, Shandong 250012, P.R. China; 2 Department of Obstetrics and Gynecology, Jiaozhou Central Hospital of Qingdao, Qingdao, Shandong 266300, P.R. China

**Keywords:** struma ovarii, pseudo-Meigs’ syndrome, CA 125, mature teratoma, ascites, pleural effusion

## Abstract

Struma ovarii is an uncommon ovarian teratoma comprised predominantly of mature thyroid tissue. The combination of pseudo-Meigs’ syndrome, and elevation of CA 125 to the struma ovarii is a rare condition that can mimic ovarian malignancy. We reported a case of benign struma ovarii, presenting with the clinical features of advanced ovarian carcinoma: complex pelvic mass, gross ascites, bilateral pleural effusion and markedly elevated serum CA 125 levels. The patient underwent total abdominal hysterectomy and bilateral salpingo-oophorectomy. Ascites and pleural effusion were not evident and the CA 125 levels returned to normal following surgical excision. A systematic review of reported cases of coexistent benign struma ovarii, pseudo-Meigs’ syndrome and elevated serum CA 125 was performed. Struma ovarii accompanied by pseudo-Meigs’ syndrome and elevated serum CA 125 should be considered in the differential diagnosis of ovarian epithelial cancer.

## Introduction

Struma ovarii is a rare ovarian neoplasm consisting almost exclusively of mature thyroid tissue (>50%) derived from germ cells in a mature teratoma (1). Few of these cases undergo malignant transformation (2). Meigs’ syndrome refers to a solid benign ovarian neoplasm, such as fibroma or thecoma accompanied by ascites and hydrothorax which are required to completely resolve following removal of the tumor (3). Pesudo-Meigs’ syndrome is often characterized by pleural effusion and ascites caused by a pelvic tumor other than an ovarian fibroma. Rare cases of ovarian tumors have been associated with pseudo-Meigs’ syndrome, such as struma ovarii tumors, mucinous or serous cystadenomas, germ cell tumors and ovarian metastasis from colon and gastric cancers (2). When coexisting with pesudo-Meigs’ syndrome and elevation of CA 125, struma ovarii is highly suspected as an ovarian malignancy. Struma ovarii mimicking advanced ovarian carcinoma can cause difficulties in preoperative diagnosis (1). Diagnosis of struma ovarii can only be made by conducting histopathology (4). The present study focused on a patient presenting with struma ovarii, who was initially thought to have an ovarian malignancy prior to surgery based on clinical, radiological findings and raised CA 125 levels. However, the frozen section and final histopathology reports revealed benign struma ovarii. A systematic review of the related literatures on struma ovarii presenting as pseudo-Meigs’ syndrome with elevated serum CA 125 was also conducted. Written informed consent was obtained from the patient.

## Case report

On April 3, 2014, a 52-year-old, Chinese female, premenopausal, gravida 3, para 1, was admitted to the United Hospital of Dezhou (Dezhou City, China), complaining of oppression in chest and shortness of breath for 5 days. The patient’s previous menstrual period was March 31, 2014. The patient did not complain of any pain or changes in micturition or bowel movements. The patient’s medical history included surgery for an ovarian tumor 26 years previously and surgery for a broad ligament tumor 10 years previously. Non-enhanced CT imaging of the chest showed bilateral pleural effusions, particularly the right thoracic cavity. Marked ascites, and a large solid and cystic mass (65×56×69 mm) in the right ovary were detected by pelvic ultrasound. On April 8, 2014, the patient was subsequently transferred to the Department of Gynecology of Qilu Hospital, Shandong University (Jinan, China).

On admission, the patient was found to have ascites and bilateral pleural effusion. The gynecological examination revealed a mass in the right adnexal region with a normal-sized mobile uterus. Abdominal and pelvic ultrasound confirmed the presence of ascites and a large irregular, cyst-solid-mixed mass in the right ovary, ~75×56 mm in size. CT scan of the chest, abdomen, and pelvis revealed bilateral lung basal atelectasis with a large right pleural effusion, gross ascites, and a large loculated complex cystic pelvic mass. There was no evidence of enlarged lymph nodes. To alleviate symptoms and aid in the diagnosis, thoracentesis was performed to yield straw-colored fluid (800 ml) consistent with an exudative process. There was approximately 2,000 ml pleural effusion. Paracentesis yielded an exudate (2,200 ml), found to be negative for malignant cells and mycobacterium tuberculosis. Cytological examination of the fluid revealed benign mesothelial cells and a few lymphocytes without malignant cells. The serum CA 125 level was 1,289 U/ml (normal value <35 U/ml). The AFP and CEA levels were within normal range. Liver function tests were also within normal limits.

The patient was arranged for an exploratory laparotomy through a vertical supraumbilical midline excision for diagnostic and therapeutic purposes. The patient was found to have ascites and 1,000 ml of straw-colored fluid was drained upon entrance to the peritoneal cavity. Extensive adhesions between posterior/left wall of uterus, left ovary, oviduct and intestinal canal as well as its surrounding tissues were identified. A 7×5 cm mixed cystic-solid neoplasm was found to arise from the right ovary. The left ovary, the two fallopian tubes, the uterus, diaphragm, bowel, and omentum appeared to be free of disease. There was no evidence of enlarged lymph nodes or metastatic lesions. The right tube and ovary were removed for frozen section and it was suggestive of cystic mature teratoma with a large component of thyroid. The patient and family member insisted on a hysterectomy and left salpingo-oophorectomy, which were performed.

**Figure 1:**
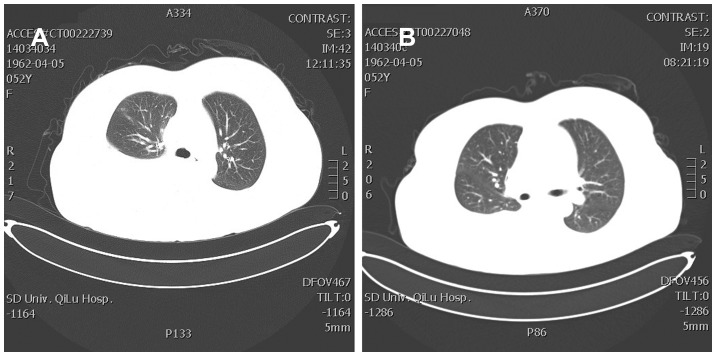
Chest computed tomography: comparison of pre- and post-operation. (A) Prior to operation and (B) seventh day post-operation.

**Figure 2:**
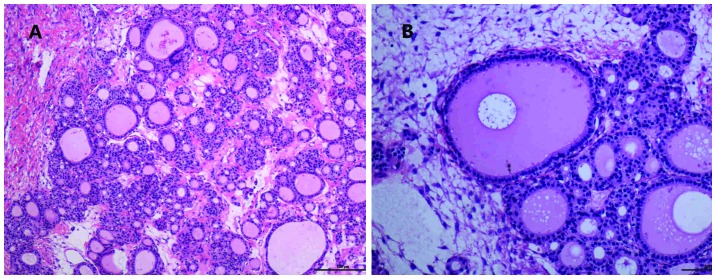
Photomicrograph showing multiple benign colloid-filled thyroid follicles with compressed ovarian stroma in the periphery. (A) Low-power view(magnification ×10); (B) high-power view (magnification ×20).

Thyroid function tests were not performed prior to surgery as the struma ovarii was not taken into consideration. Following the diagnosis, thyroid function tests were obtained on the third postoperative day. The results of the tests revealed normal thyroid function with serum FT3 levels at 2.05 pg/ml (1.8–4.6 pg/ml), FT4 levels at 17.79 pmol/l (12–22 pmol/l) and TSH levels at 2.9 uIU/ml (0.27–4.2 uIU/ml). Mild hypoalbuminemia was observed. On the sixth day post-operation the level of CA 125 was decreased to 609.6 U/ml. On the seventh day post-operation another chest CT was taken, examined and compared with the pre-operative one (Fig. 1). The final pathology revealed right struma ovarii with benign thyroid tissue confined to the ovary (Fig. 2). The left ovary, the uterus and bilateral fallopian tube were histologically unremarkable. The rapid regression of effusions was demonstrated following excision of the neoplasm. The patient recovered uneventfully and was discharged on day 12 post-operatively. Recovery was rapid, with no evidence of re-accumulation of the pleural effusions or ascites. At 8 weeks follow-up, the patient was clinically well, with no evidence of disease on physical examination and normal CA 125 levels (6.5 U/ml).

## Discussion

Struma ovarii is an uncommon benign neoplasm of ovary that usually presents with asymptomatic mass and is difficult to diagnose prior to surgery. Ascitic fluid is identified in 20% of cases of struma ovarii (5). Struma ovarii has been associated with pseudo-Meigs’ syndrome in 5% of cases (6). The detailed mechanism of ascites and pleural effusions is obscure. Potential explanations include: irritation of the peritoneum by the tumor, obstruction of the lymphatics, toxins and release of inflammatory products, hypoalbuminemia, and discrepancy between the arterial supply and the venous and lymphatic drainage (7). Regarding the mechanism of pleural effusions, dye test results have shown that these effusions are likely to originate from the peritoneal fluid by mechanical transfer through diaphragmatic openings (7).

Serum tumor markers are useful in determining the potential malignancy of a mass. CA 125 is a classical tumor marker that is effective in the surveillance of treated epithelial ovarian cancers. However, CA 125 has poor specificity in the diagnosis of epithelial ovarian cancers, as its elevation may also be associated with other malignancies and benign, physiological states, including pregnancy, endometriosis and menstruation (8). Elevated CA 125 accompanied by Meigs’ syndrome is a rare clinical condition that was reported in only 27 cases (4). The exact reason for the elevated CA 125 in Meigs’ and pesudo-Meigs’ syndrome remains unclear. A possible explanation proposed by Mui *et al* (4) is the irritation and subsequent inflammation of pleura and peritoneum surface produced by the presence of free fluid in these spaces.

A postmenopausal female presenting with a pelvic mass, ascites, pleural effusions, and elevated CA 125 levels generally is highly indicative of a malignant process. Few cases of struma ovarii accompanied with pseudo-Meigs’ syndrome, elevated CA 125 have been described. We performed a systematic review of related studies obtained from PubMed by using a combination of free words and MeSH. The search was not limited by publication time or English literature. Ten case reports of struma ovarii combined with pesudo-Meigs’ syndrome and elevated CA 125 level were identified (Tables I and II). We report an eleventh case in the literature with struma ovarii associated with pseudo-Meigs’ syndrome and elevated CA 125. Struma ovarii occurs mainly during the 5th-6th decade of life. Almost 73% (8/11) of cases were postmenopausal women. The average reported size of the tumor was 10 cm in the large dimension, mostly unilateral, with only 9.1% being bilateral, with a right-side predominance and CA 125 levels that were moderately elevated [124.9 U/ml (9)] or extremely elevated [3,803 U/ml (10)]. Approximately 36.4% (4/11) cases coexisted with thyroid disease, 50% for hypo- and hyperthyroidism, respectively. All the cases except those reported by Rana *et al* (11) were initially suspected to be a malignant tumor. Complete remission of the ascites, hydrothorax, and CA 125 was obtained following surgery without any adjuvant therapy. Positive prognosis for all the cases was reported.

A number of unique features were identified in the patient. Firstly, she presented with the sudden onset of large pleural effusions. Secondly, she was premenopausal, her age being younger than that of patients in which the majority of these tumors occur. The rapid onset of oppression in chest and shortness of breath, sinister ultrasound findings (marked ascites, a 65×56×69 mm solid and cystic mass to the right adnexal region) and a significantly elevated CA 125 level were highly suspicious for an ovarian malignancy. Struma ovarii accompanied by pseudo-Meigs’ syndrome and elevated serum CA 125 should be considered in the differential diagnosis of ovarian epithelial cancer.

## Figures and Tables

**Table I:** General characteristics of reported struma ovarii associated with pseudo-Meigs’ syndrome and elevated CA 125 levels.

Author	Year	Age (years)	Menstruation	Tumor size (cm)	Unilateral or bilateral	CA 125 (U/ml)	Ascites volume (ml)	Pleural effusions (ml)	Refs.
Bethune *et al*	1996	62	Postmenopause	9×5×5	Right	1621	Small amount	3500	(12)
Long *et al*	2001	53	Postmenopause	15×11×7	Left	540	4100	NA	(9)
		78	Postmenopause	12×10×5.2	Left	124.9	NA	NA	
Huh *et al*	2002	65	Postmenopause	5×4×4	Right	402	20000	NA	(13)
Loizzi *et al*	2005	65	Postmenopause	7×7	Right	161	Few liters	Large amount	(5)
Obeidat *et al*	2007	52	Postmenopause	10×15×8	Right	149	4000	NA	(14)
Mitrou *et al*	2008	55	Postmenopause	22×23×10	Left	3803	8000	NA	(10)
Paladini *et al*	2008	42	Premenopause	11×7.3×8	Right	2548	8000	NA	(15)
Rana *et al*	2009	70	Postmenopause	7.5×5.5×4	Bilateral	284	NA	NA	(11)
Jiang *et al*	2010	46	Premenopause	20×18×15	Right	1230.9	6000	NA	(1)
Present	2014	52	Premenopause	7×5	Right	1289	1000	2000	

NA: Not available.

**Table II:** Clinical symptoms, treatments, coexisting thyroid disease of reported struma ovarii associated with pseudo-Meigs’ syndrome and elevated CA 125 level.

Author	Clinical symptoms	Treatments	Coexisting thyroid disease	Refs.
Bethune *et al*	Acute shortness of breath and ascites	Total abdominal hysterectomy and bilateral salpingo-oophorectomy	Absent	(12)
Long *et al*	Abdominal distension and weight loss	Total abdominal hysterectomy, bilateral salpingo-oophorectomy and infracolic omentectomy	Absent	(9)
	Abdominal distension, ielus and weight loss	Total abdominal hysterectomy and bilateral salpingo-oophorectomy	Absent	
Huh *et al*	Abdominal distension, dyspnea	Total hysterectomy and bilateral salpingo-oophorectomy and appendectomy and omental biopsy	Hypothyroidism	(13)
Loizzi *et al*	Dyspnea and diffuses abdominal pain	A right salpingo-oophorectomy	Hyperthyroidism	(5)
Obeidat *et al*	Shortness of breath and marked ascites	A total abdominal hysterectomy, bilateral salpingo-opherectomy and omentectomy	Absent	(14)
Mitrou *et al*	Large pelvic mass, marked cachexia, ascites	A total abdominal hysterectomy with bilateral salpingo-oophorectomy, infracolic omentectomy, and lymph node sampling	Hypothyroidism	(10)
Paladini *et al*	Ascites, fever, diarrhea, vomiting and significant weight loss	Right salpingo-oophorectomy	Hyperthyroidism	(15)
Rana *et al*	Progressive abdominal distention and breathlessness	Total abdominal hysterectomy with bilateral salpingo-oophorectomy and partial omentectomy	Absent	(11)
Jiang *et al*	Fatigue, anorexia, and abdominal swelling	Total abdominal hysterectomy with bilateral salpingo-oophorectomy	Absent	(1)
Present	Oppression in chest and shortness of breath	Total abdominal hysterectomy with bilateral salpingo-oophorectomy	Absent	

NA, not available.
